# Longitudinal measures of pupil barycenter and estimation of angle kappa in young children

**DOI:** 10.1111/opo.70040

**Published:** 2025-11-04

**Authors:** Deepa Dhungel, Aidan McCullough, Fuensanta A. Vera-Diaz

**Affiliations:** New England College of Optometry, Boston, Massachusetts, USA

**Keywords:** angle kappa, children, myopia, pupil barycenter

## Abstract

**Purpose:**

The angle between the visual axis and the pupillary axis (angle kappa), and its approximation (angle lambda–between the line of sight and the pupillary axis), are reported to be larger in shorter eyes. However, there is only one previous study of these angles in children, and no longitudinal studies. This study evaluated angle kappa in young children over a 3-year period.

**Methods:**

Pupil barycenter measures (pupil centre relative to the corneal apex) were taken with an optical biometer and used to estimate angle kappa using the Pythagorean theorem. Both eyes of young children (6 to ≤8 years at baseline) with functional emmetropia were measured biannually for 3 years (seven visits) following complete cycloplegia.

**Results:**

The pupil barycenter was located inferior nasal to the corneal apex in most eyes, with a median absolute angle kappa of 5.01° ± 2.54° (0.33 ± 0.15 mm). There was no significant interocular difference in this angle. Correlations of angle kappa with axial length (AXL) and refractive error at baseline were not significant (both *p* > 0.05), with a trend of a smaller angle kappa in longer eyes. The rate of decrease of angle kappa (slope) was significant for the right eye, as the rate of increase in AXL increased (*r* = 0.33, *p* < 0.01), but not in the left eye. The rate of change in AXL and refractive error did not show a significant correlation with the rate of change in angle kappa (*p* > 0.05).

**Conclusions:**

Angle kappa appears to be independent of AXL or refractive error in these young children with a limited range of eye sizes. However, angle kappa may decrease at a higher rate in eyes with a greater rate of increase in AXL over the course of 3 years.

## INTRODUCTION

Angle kappa is defined as the angle between the visual axis and the pupillary axis of the eye.^1^ The visual axis is the imaginary line that passes through the nodal point of the eye, connecting the fixation point and the fovea. The pupillary axis is the imaginary line perpendicular to the anterior corneal surface, passing through the centre of the entrance pupil. Angle kappa is nearly identical to angle lambda, that is, the angle between the pupillary axis and line of sight.^2–4^ A schematic showing angle kappa and the corresponding axes is shown in Figure 1.

Measurement of angle lambda, or kappa, is clinically important in the management of strabismic patients,^5,6^ and the presence of an abnormal angle kappa would either modify the aesthetical appearance of the ocular deviation or create one.^7–9^ It is also an important metric in refractive surgery^10^ and determining intraocular lens (IOL) placement,^11^ as a large angle kappa can lead to alignment errors, with potential under correction.^12^ Knowledge of angle kappa is also important in optical measurements, including peripheral aberrations.^13,14^ Although the pupillary axis can be identified clinically, because the visual axis is a line in reference to an imaginary landmark inside the eye (the nodal point), angle kappa is not usually measured but rather estimated in close proximity to angle lambda using clinical devices such as optical biometers or corneal topographers.^2,6,15,16^ Clinically, angle kappa is typically identified by the displacement of the corneal light reflex (the reflection on the cornea, defined as the subject-fixated coaxially sighted corneal light reflex) from the centre of the pupil.^1,17^ Angle kappa is classified clinically as positive if the co-axial corneal light reflex is on the nasal aspect, and negative if the reflex is temporal to the pupil centre in the right eye.^1,5,17–21^ A small positive angle kappa of about 5° (~0.30 mm) is considered typical in adults, as the fovea is slightly temporal to the centre of the posterior pole of the eye.^2,12,17,22^ Angle kappa has been reported to be larger in shorter eyes, for example, in adults with hyperopia compared with myopes, or in younger compared with older adults.^23^

A previous study reported that the average estimated angle kappa in young children aged 3–15 years was 0.27 ± 0.22 mm, with the pupil centre being located in the inferior temporal quadrant.^24^ They noted no significant effect with the age of the children. However, there are no previous longitudinal studies of angle kappa in young children during their development. In addition, potential differences in angle kappa before the development of myopia may be an intrinsic factor that might predispose young children to develop myopia.^25^ This study used a commercially available optical biometer to assess longitudinal measures of pupil barycenter offset and estimates of angle kappa in young children.

## METHODS

### Subjects

Participants (*n* = 102) were young children (mean age 7.49 ± 0.96 years at baseline) from the Greater Boston area enrolled in the PICNIC (Preventing Myopia: Investigating Contributing Factors to Nearsightedness in Children) longitudinal study at the New England College of Optometry. Participants had functional emmetropia (emmetropia or low hyperopia, <+2.50 and with no visual consequences, e.g., they did not need optical correction) at the baseline visit. The inclusion and exclusion criteria, as well as the PICNIC study design, are available elsewhere.^25–27^ In brief, the general inclusion criteria, as determined at the baseline visit, were: (1) between 6 and <9 years of age; (2) uncorrected distance visual acuity (VA) of +0.10 LogMAR or better in each eye and (3) cycloplegic refractive error (spherical equivalent, M) not less than −0.25 D and no more than +2.50 D in the right eye, with ≤0.75 D of astigmatism in either eye and ≤0.75 D of anisometropia. Refraction was determined under cycloplegia (two eye drops of 1% Cyclopentolate, instilled 5 min apart into each eye) using open-field autorefraction (Grand Seiko WAM-5500, grandseiko.com/). After the initial baseline visit, subjects who met all the inclusion criteria were asked to return every 6 months (±2 weeks) for 3 years, for a total of seven visits. Of the 102 children enrolled in PICNIC, data for 92 children who completed a minimum of three visits are included in this report. Children were classified at baseline (Visit 1) as either being at low risk (LR, *n* = 48) or high risk (HR, *n* = 44) for myopia, based on their cycloplegic refractive error (M) and parental myopia. Throughout the study, children were classified into a myopia group when their cycloplegic open-field autorefraction M values in the right eye became ≤−0.25 D.^25–27^ Out of the 44 children included in the HR group, 14 developed myopia during the study duration, whereas no children from the LR group developed myopia during the course of the study. More details on the study design can be found in previous reports^25–27^ and Table A1.

### Procedures

Participants completed a standard clinical assessment, including cycloplegic open field autorefraction and ocular biometry, at each study visit as a part of the PICNIC study. Other tests were also performed, including static and dynamic accommodative responses, central and peripheral ocular aberrations, psychophysical peripheral blur discrimination, contrast sensitivity, retinal responses to simulated blur with an electroretinogram and wide-field posterior segment optical coherence tomography measurements. These are experimental tests within the PICNIC study and are reported elsewhere but are not presented in this manuscript.^25–27^

Biometric readings were taken using a Haag-Streit Lenstar 900 biometer (haag-streit-usa.com), following complete cycloplegia. Five measurements were taken for each eye and averaged. Pupil barycenter (PCx and PCy) values were obtained from these biometry measurements. PCx and PCy are the horizontal and vertical distances from the centre of the entrance pupil to the corneal vertex, respectively. A positive and negative PCx value represents a nasally or temporally located pupil centre relative to the corneal apex, respectively. Similarly, positive and negative PCy values indicate a superior or inferior location of the pupil centre relative to the corneal apex, respectively. The corneal vertex is seen as the first Purkinje image in the Lenstar image as shown in Figure 2a. The estimated angle lambda or the approximate angle kappa was calculated using the chord μ calculated using the Pythagorean theorem:

$$\text{Angle}\;\text{kappa}=\tan^{-1}(\frac{\text{chord}\;\mu }{\text{ACD}})$$

where, chord $\mu =\sqrt{{\text{PCx}}^{2}+{\text{PCy}}^{2}}$ and ACD is the anterior chamber depth in mm.

Figure 2b shows the schematic representation of angle kappa components PCx and PCy, in relation to the corneal apex. Chord μ is the distance between the pupil barycenter and the corneal apex (pupillary axis). By definition, angle kappa is the angle between the pupillary axis and the visual axis, while angle lambda is the angle between the pupillary axis and the line of sight.^1,13,16^ For distance fixation, the line of sight and the visual axis are almost aligned.^3,4,16^ Therefore, although the distance between the pupil barycenter and the line of sight was estimated, it will still be referred to as angle kappa, rather than angle lambda, as this is widely accepted in the literature.^13^

### Data processing and analyses

PCx, PCy and axial length (AXL) were measured using the biometer. The estimated value of angle kappa was computed as described previously using the Pythagorean theorem. The *M* value of the cycloplegic refractive error, that is, $S+\frac{1}{2}C$ (where *S* is the spherical component and *C* is the cylindrical component) was also used in the data analysis. The values obtained at the first visit were used as the baseline measure. To assess changes over time, the slope of a simple linear regression model between each of the three measures was used, namely angle kappa, AXL and *M*, as well as the visit number, to estimate the rate of change in these measures per visit.

Statistical analysis was carried out using R software (r-project.org). Since the data were not normally distributed, non-parametric statistical tests were used. Interocular differences were assessed using a paired Wilcoxon signed-rank test. Spearman correlation analyses were performed to assess the correlation between both AXL (obtained with cycloplegic Lenstar measures) and M with the magnitude of angle kappa.

There was significant variability in the results across visits for individual subjects, in addition to intrasubject variability. To enhance the reliability of the data, systematic data filtering was applied, in which data for a subject across all seven visits were pooled for each eye separately and PCx and PCy values that were greater than the sum of the third quartile, 1.5 × IQR or less than the difference between the first quartile and 1.5 × IQR were discarded. Approximately 18% of the data was discarded using this method. After filtering, subjects with data for at least three visits were included in the subsequent analyses.

## RESULTS

The findings are summarised in Table 1. The results for baseline (Visit 1) pupil barycenters (PCx, PCy) for each eye are shown in Figure 3. The majority of the PCx values were negative for the right eye and positive for the left eye, and the majority of the PCy values were negative, meaning the pupil barycenter was located inferior temporal to the corneal reflex in most cases. This corresponds to an inferior nasal angle kappa, clinically referred to as a positive angle kappa with a vertical component. The baseline PCx values did not show a significant difference between the right (median ± IQR = −0.28 ± 0.16 mm) (Figure 3) and left eyes (median ± IQR = +0.29 ± 0.14 mm) (Figure 3), absolute values on the Wilcoxon signed-rank test (*W* = 942.50, *p* = 0.84). Absolute values of the coordinate PCx were used for the median and Wilcoxon signed-rank test as the signs indicated the location (i.e., direction) from the corneal apex. When considering the refractive risk group (i.e., LR, HR or Myopia), the pupil barycenter did not show any separate clustering for either eye, indicating no significant difference between the three groups (see Figures A1 and A2) at any of the visits. The AXL measurements for each visit and refractive group are shown in Figure A3.

The rate of change of PCx (horizontal location of the pupil barycenter) with time was determined by calculating the PCx slopes across the seven visits (3 years). There was no significant change in PCx in either the right (Median slope = 7.82 × 10^−4^ ± 0.01 mm per visit) or the left eye (Median slope = −14 × 10^−4^ ± 0.01 mm per visit) over the course of the study. There was also no significant difference in the rate of change of PCx (*W* = 2089.00, *p* = 0.99) between the two eyes.

The median (±IQR) baseline AXL was 22.72 ± 0.71 and 22.71 ± 0.78 mm in the right and left eyes, respectively, with no significant difference between the two eyes (*W* = 2420.00, *p* = 0.09). The median (±IQR) baseline angle kappa absolute values were: right eye 4.97° ± 2.45°, left eye 5.32° ± 2.38°, with no significant difference between the two eyes (*W* = 924.00, *p* = 0.18). Note that the sign of the angle kappa indicates the location of the pupil barycenter but does not have numeric implications; therefore, the magnitude of angle kappa is plotted in Figure 4a.

Figure 4b shows the rate of change of angle kappa (slope) for each eye. The Median ± IQR *slope* of angle kappa in the right and left eye was −0.03 ± 0.21 and −0.03° ± 0.53° per visit, respectively. There was no significant difference in these slopes between the two eyes (*W* = 1652.00 , *p* = 0.15).

Additionally, a Spearman correlation analysis was conducted between the baseline AXL and baseline angle kappa, as shown in Figure 5. The correlation between baseline AXL and baseline angle kappa was not significant for either the right (*r* = 0.08, *p* = 0.48) or the left eye (*r* = −0.14, *p* = 0.28), although there might be a trend for smaller angle kappa values in longer left eyes.

Spearman correlations between baseline M and the baseline angle kappa are shown in Figure 6. The median (±IQR) value of M was +1.00 ± 0.78 and +0.92 ± 0.76 D in the right and left eyes, respectively. There was a small and statistically (not clinically) significant difference in M between the two eyes (*W* = 2788.00, *p* < 0.001). The correlation between baseline M and baseline angle kappa did not reach significance in the right (*r* = 0.08, *p* = 0.48) or left eyes (*r* = −0.06, *p* = 0.68) (Figure 6).

To study the change in angle kappa with the change in AXL during the 3-year duration of the study, a Spearman correlation analysis was conducted between the slopes for AXL and the magnitude of angle kappa. As expected, since AXL increased with time, the slope of AXL was always positive for both the right (Median ± IQR = 0.07 ± 0.04 mm per visit) and left eyes (Median ± IQR = 0.07 ± 0.04 mm per visit). The slope of AXL did not show a significant difference between the two eyes (*W* = 1822.00, *p* = 0.22). The correlation between the change in AXL (slope) and the change in angle kappa (slope) across the 3 years was significant in the right eye (*r* = −0.31, *p* < 0.01), but not in the left eye (*r* < 0.001, *p* = 0.99). The correlation in the right eye indicates a higher rate of decrease in angle kappa with an increasing rate of AXL in the right eye, as shown in Figure 7.

The slopes of M, for both the right (median ± IQR = −0.04 ± 0.07 D per visit) and left eyes (median ± IQR = −0.02 ± 0.07 D per visit), were small and negative, indicating the expected decrease in hyperopia over time. A significant difference in the slopes was noted between the two eyes (*W* = 1419.00, *p* < 0.01), with more change in the right eye. However, the Spearman correlation between the slope of *M* and the slope of angle kappa did not show a significant correlation in either eye (both *p* > 0.76) as shown in Figure 8.

## DISCUSSION

The results of this study showed that angle kappa can be estimated using a widely available commercial optical biometer in young children. This is the first longitudinal report on estimated angle kappa measurements in children of this age group. Measurements were obtained after cycloplegia to decrease possible variations in anterior chamber depth and therefore angle kappa due to accommodation during the testing. The obtained values are similar to those from earlier studies in adults and older children.^2,12,17,28^ Consistent with Ye et al.^24^ and previous studies in adults,^2,12,17,20–22^ the pupil barycenter was located in the inferior temporal quadrant relative to the corneal apex, indicating that the eyes were rotated nasally about the y-axis and upwards about the x-axis.

The median ± IQR of the absolute angle kappa values in the current study was 0.33 ± 0.15 mm (5.01° ± 2.54°) for both eyes, which is similar to the mean value (0.27 ± 0.22 mm) found in children by Ye et al.^24^ Similarly, in agreement with their findings, no significant correlation was found between angle kappa and either AXL or refractive error. Previous studies in adults have shown a significant difference in the angle kappa values between the two eyes,^6,12,29,30^ with a larger angle kappa in the left compared with the right eye.^6,28,29^ No significant interocular difference in angle kappa values was found at baseline, and the slope of angle kappa over time did not differ between the two eyes, indicating that neither angle kappa nor the rate of change in values showed interocular differences in young children with functional emmetropia. Note that these children are not representative of the entire population, as children with refractive errors or children with strabismus were not included in this investigation.

Some studies have shown a significant correlation between angle kappa and AXL, with a decrease in the angle with increasing AXL,^23,31,32^ while others have reported no significant correlation between angle kappa and AXL.^17,24,33^ In the current investigation, while the baseline AXL did not show a significant correlation with angle kappa, a significant correlation was observed between the change in angle kappa over time and the change in AXL in the right eye, but not in the left eye. It is worth noting that the right eye was significantly more hyperopic than the left eye at baseline, indicating that M and AXL in the two eyes change at different rates. Therefore, we speculate that the higher rate of decrease in angle kappa with a larger increase in AXL over time observed only in the right eye could be due to that eye being ‘refractively behind’, that is, more hyperopic and hence showed a larger change in AXL with time. Moreover, since the subject pool had a limited range of AXL, it is likely that possible shifts in angle kappa with the changes in AXL have not been fully captured here. Future studies should consider including subjects with a wider range of AXLs and refractive errors.

It is also known that angle kappa is larger in adults with shorter eyes and hyperopia compared with those with longer eyes and myopia.^15,23,31,34,35^ In the current group of young children, there was no significant correlation between refractive error and baseline angle kappa, also likely due to the small range of refractive errors included (emmetropia to low hyperopia). The baseline refractive error in the present study was functional emmetropia, and only 14 out of the 92 subjects eventually developed myopia during the course of the investigation. Due to the limited number of myopic subjects and narrow range of refractive error distribution, conclusions regarding the effect of refractive status on angle kappa cannot be drawn. Additionally, the biometric measurements were not taken at the same time of the day at each visit. Since AXL shows diurnal changes,^36–41^ the changes in AXL might have been captured better if the measurements were taken at the same time of the day at each visit.

The findings of the study demonstrate that angle kappa can be readily estimated using the pupil barycenter distance measured with an optical biometer, and the pupil barycenter is located infero-temporally in young children. Given that optical biometers were not readily available in strabismus clinics in the past, previously proposed methods to measure angles kappa or lambda in paediatric populations used photographs of children’s eyes and approximation formulas.^8^ These techniques have limitations related to the camera’s position and the need for complex calculations. They also assumed a standard corneal diameter and anterior chamber depth, both of which, particularly the anterior chamber depth, vary significantly in the eyes of young children with or without myopia. In addition, the corneal vertex was assumed to be in the same location as the corneal intersection of the line of sight. With the increasing availability of optical biometers in paediatric clinical practice for the management of myopia, their use would be beneficial for these purposes.

Angle kappa appears to be independent of AXL or refractive error, indicating that for this group of young children with a limited range of eye sizes, AXL might not result in a change in angle kappa. However, a higher rate of increase in AXL may have resulted in a larger rate of decrease in angle kappa, as indicated by the significant correlation between the rate of change of both angle kappa and AXL over time.

## Figures and Tables

**FIGURE 1:**
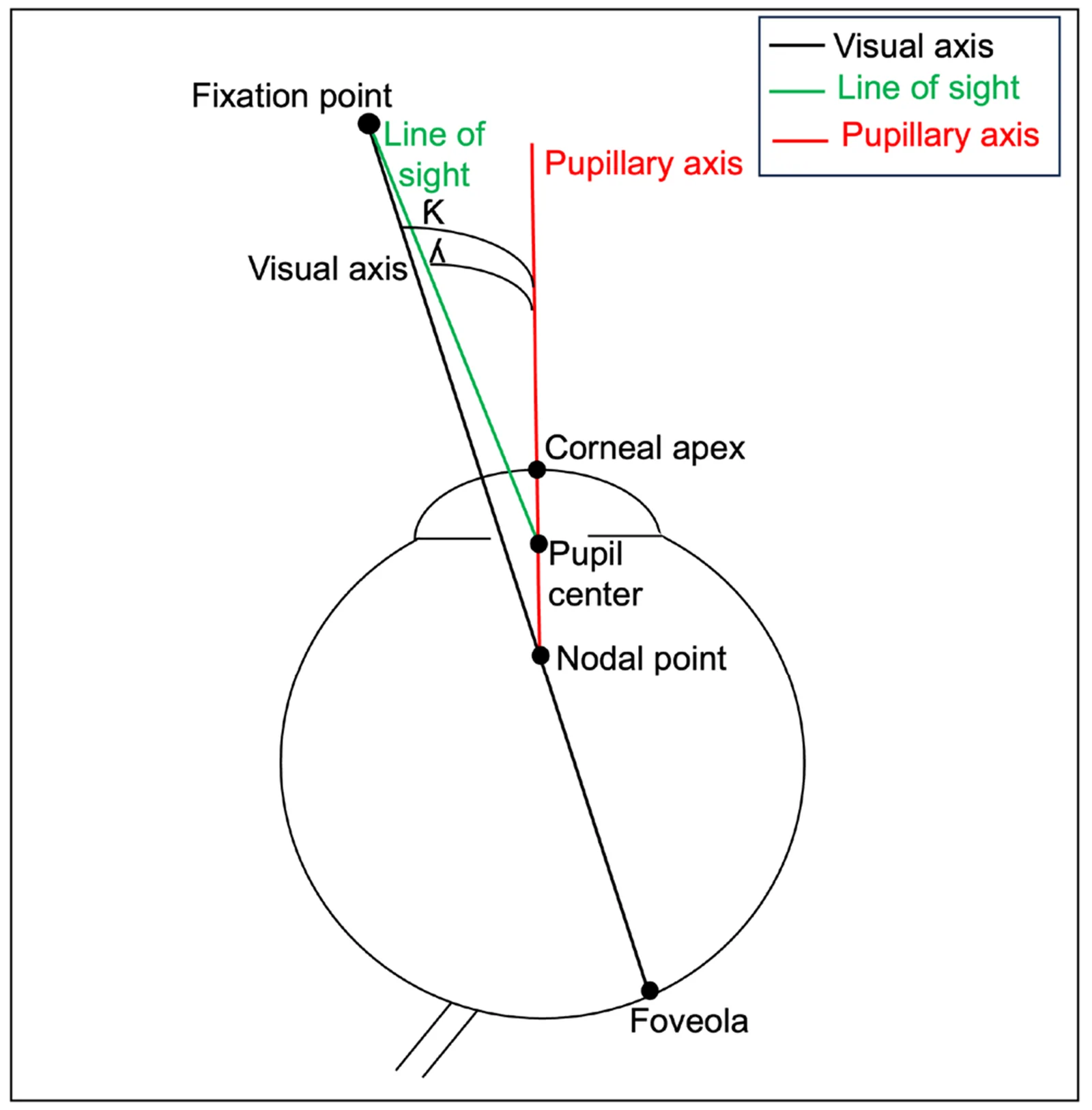
Definition of ocular angles lambda (ʎ) and kappa (κ) and their axes for the right eye. Angle kappa represents the angle between the pupillary axis and the visual axis.

**FIGURE 2:**
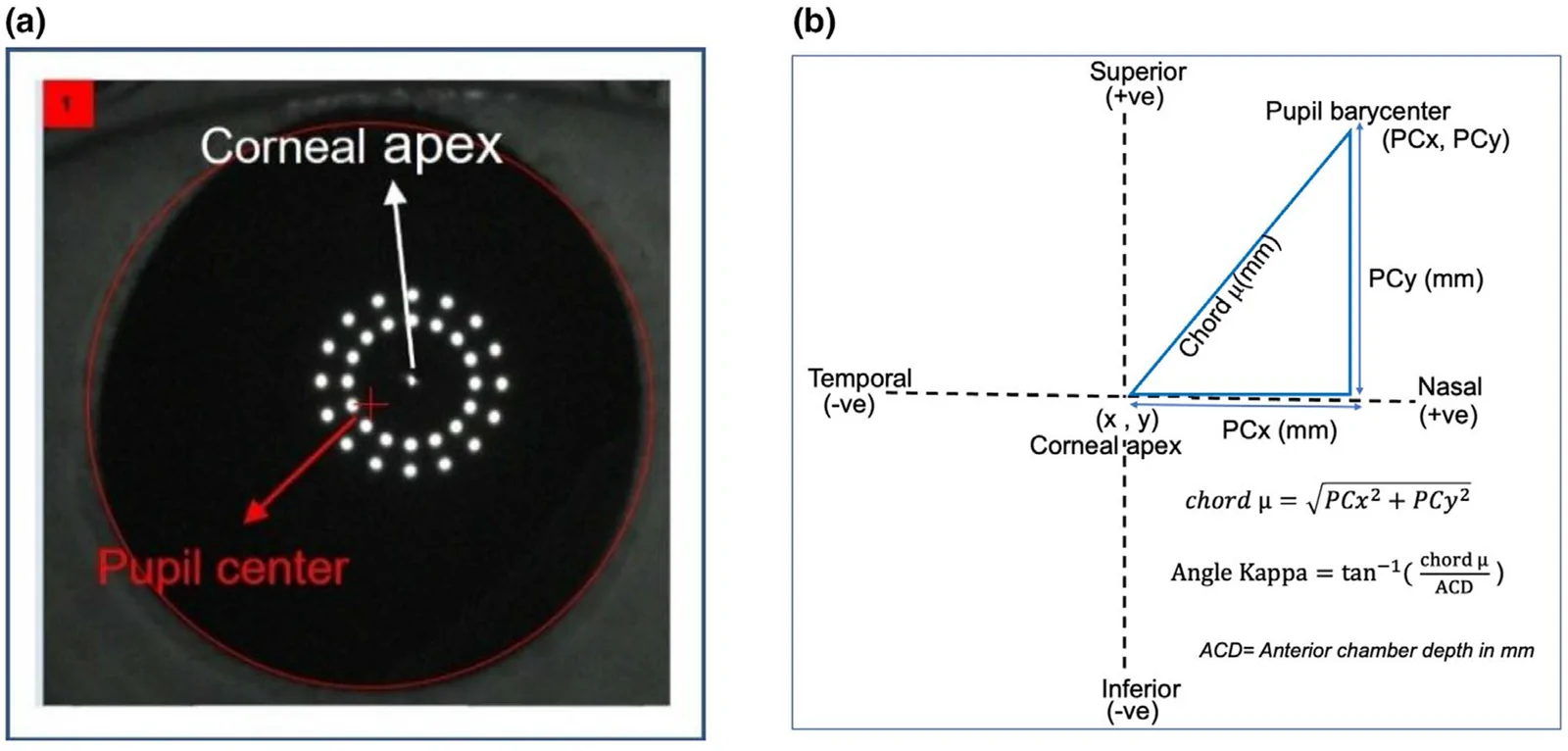
(a) Lenstar image of a subject’s right eye with marked corneal vertex and pupil centre. (b) Schematic representation of angle kappa components. PCx and PCy are the horizontal and vertical distance from the centre of the entrance pupil to the corneal vertex, respectively. Chord μ is the distance between the pupil barycenter and the corneal apex (pupillary axis).

**FIGURE 3:**
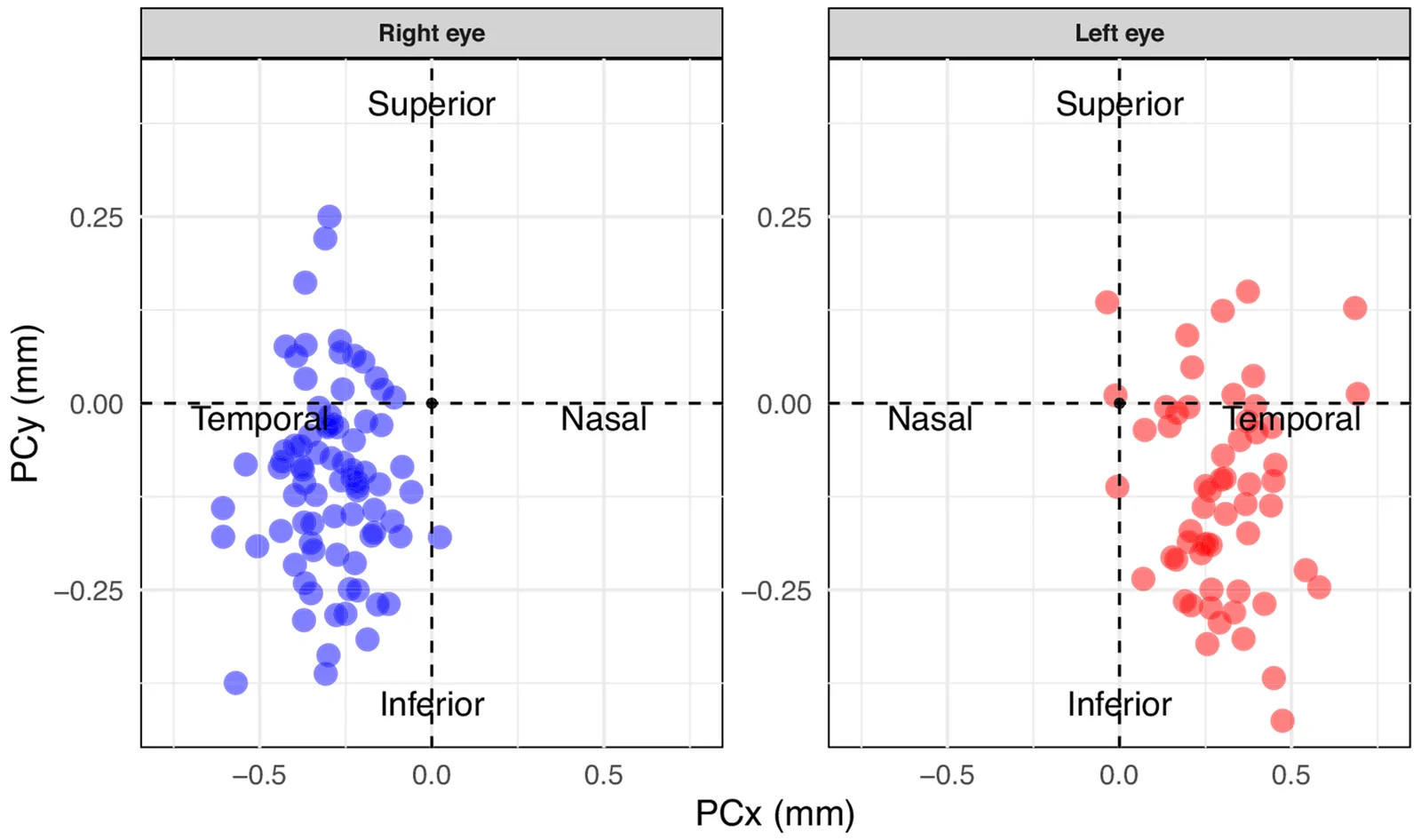
Pupil barycenter coordinate plots showing baseline PCy versus baseline PCx coordinates relative to the corneal apex (centre black dot). PCx and PCy are the horizontal and vertical distance from the centre of the entrance pupil to the corneal vertex, respectively. The blue and red dots show the right and left eye data, respectively. For the right eye, negative PCx values represent a temporal location and positive values a nasal location of the pupil barycenter in relation to the corneal apex, and vice versa for the left eye. Vertically, positive and negative PCy values represent a superior and inferior location of the pupil barycenter, respectively, for both eyes. The majority of the pupil barycenters were observed in the inferior temporal quadrant.

**FIGURE 4:**
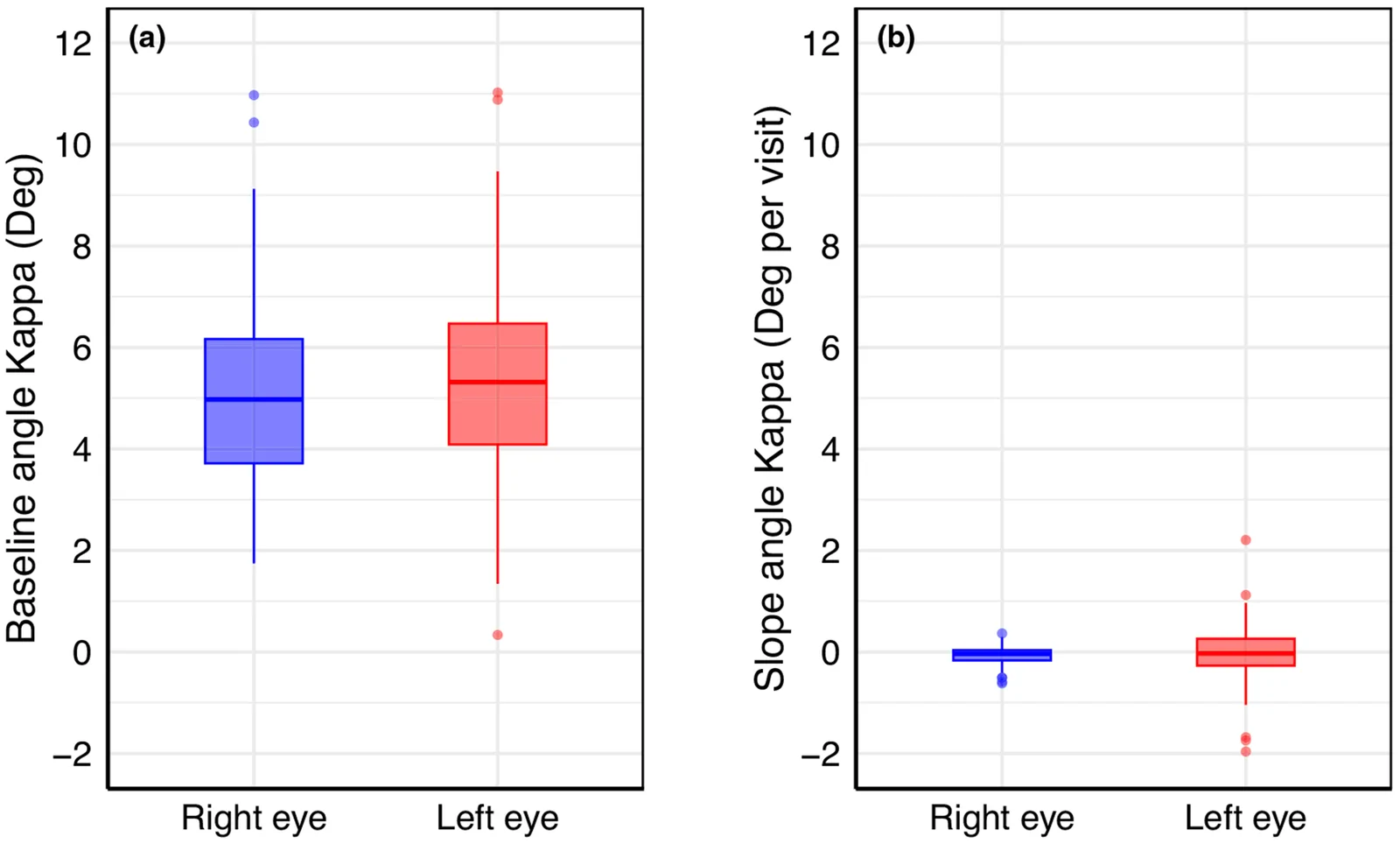
Boxplots showing the baseline estimated magnitude of angle kappa in degrees (Deg) (a), and the slope of the angle kappa values (b) for the right eyes (blue boxes) and left eyes (red boxes).

**FIGURE 5:**
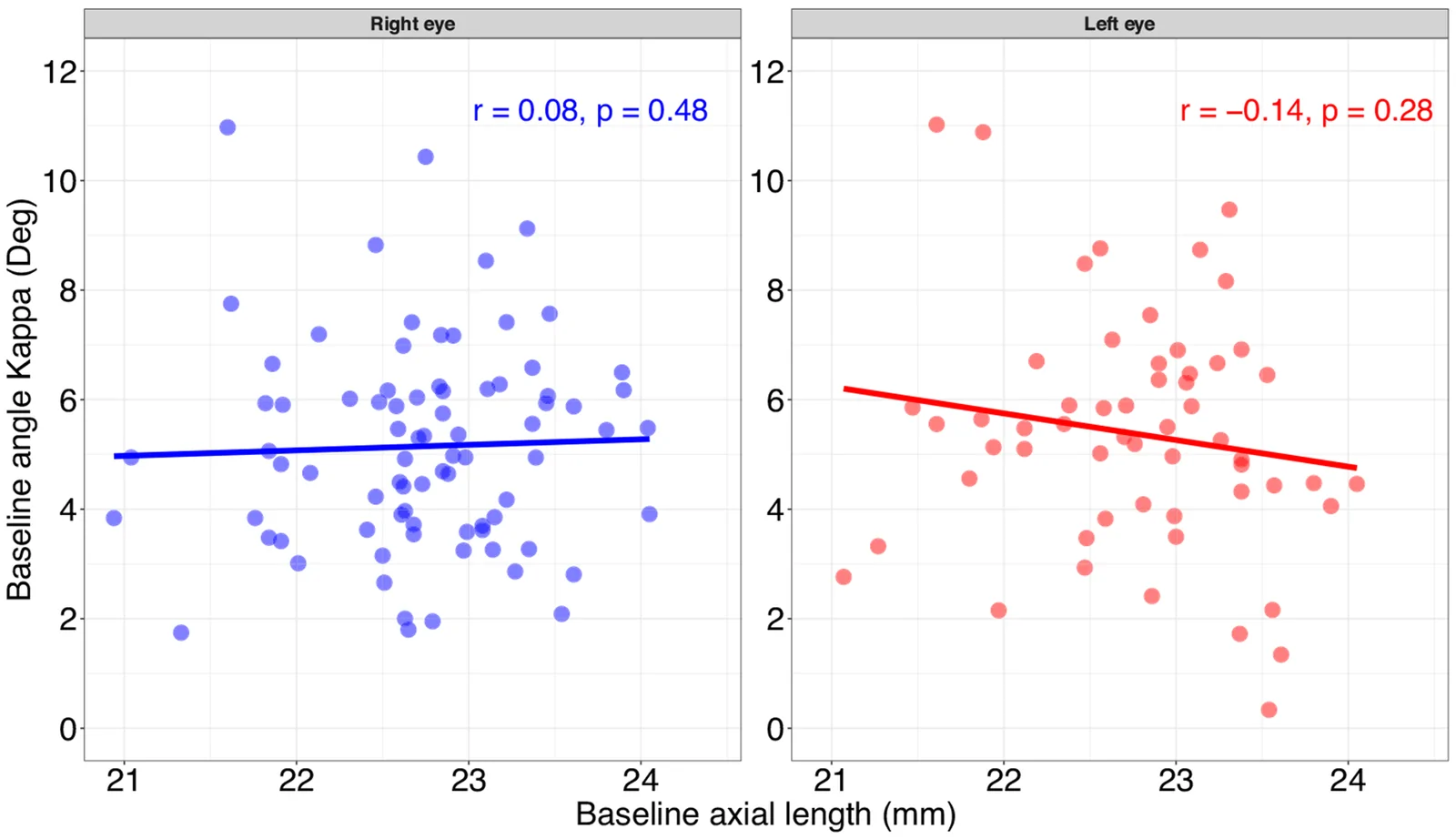
Estimated magnitude of baseline angle kappa (Deg, degrees) plotted across baseline axial length (mm) for the right eyes (blue dots) and left eyes (red dots). The angle kappa values showed an apparent decreasing trend with increasing axial length in the left eye, but the correlation was not significant.

**FIGURE 6:**
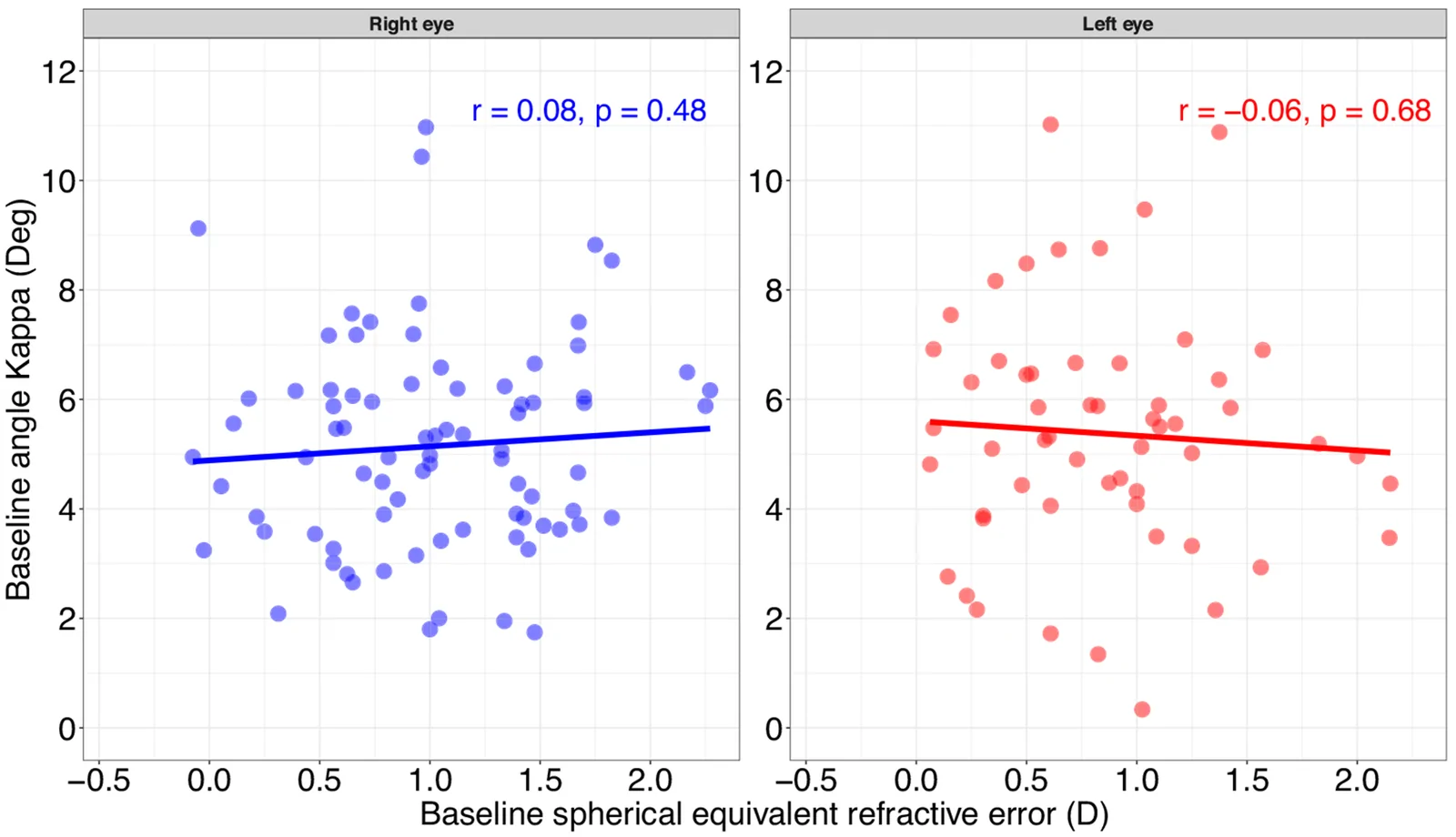
Estimated magnitude of baseline angle kappa (Deg, degrees) plotted across baseline spherical equivalent refractive error (D) for the right (blue dots) and left eyes (red dots). No significant correlations were noted between angle kappa and refractive error for either eye.

**FIGURE 7:**
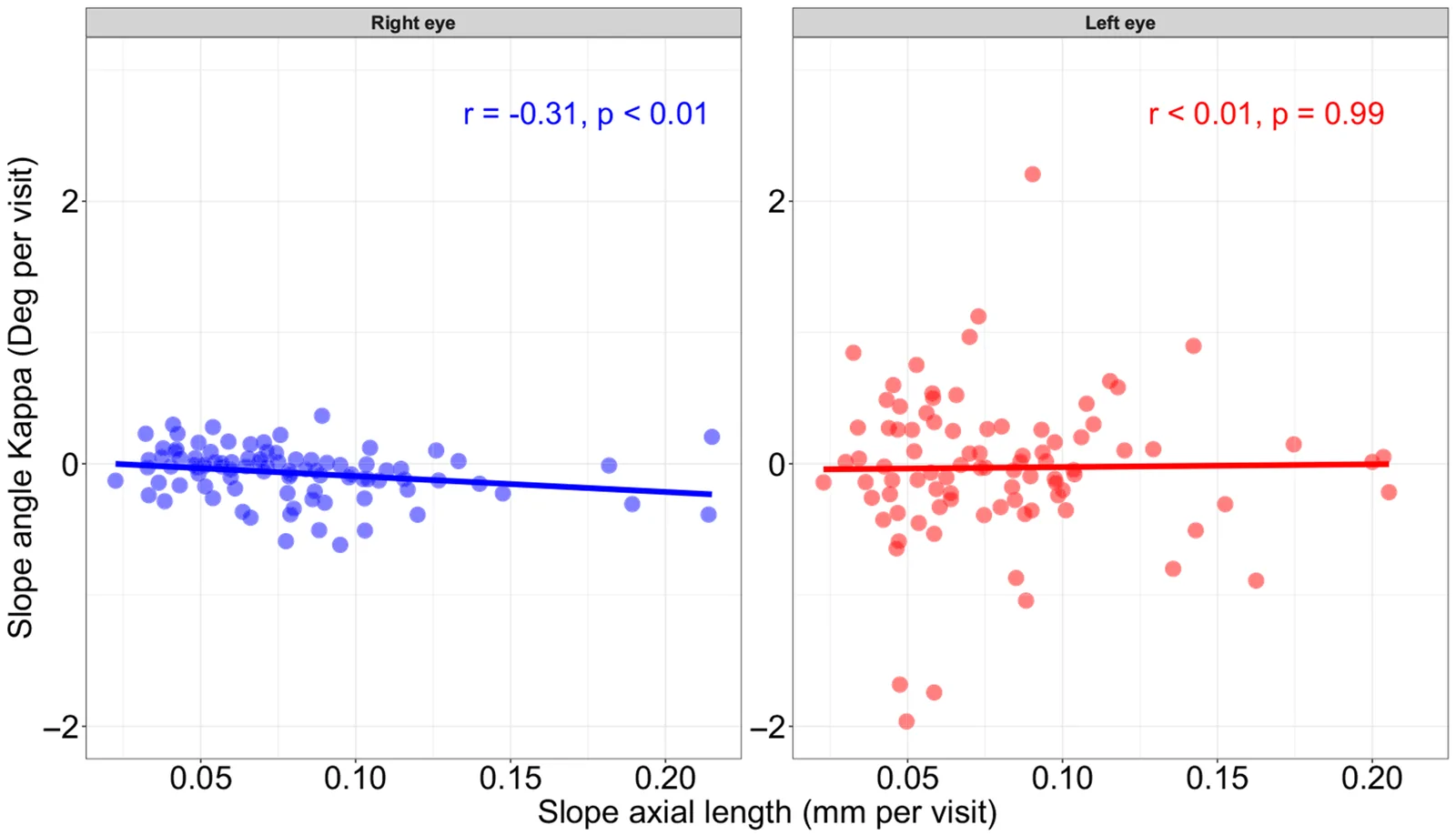
Scatterplot showing the slope of angle kappa values (in Deg, degrees), calculated as the change from Visit 1 through Visit 7, plotted against the slope of axial length (mm) for the right (blue dots) and left eyes (red dots). A significant correlation was observed in the right, but not the left eyes.

**FIGURE 8:**
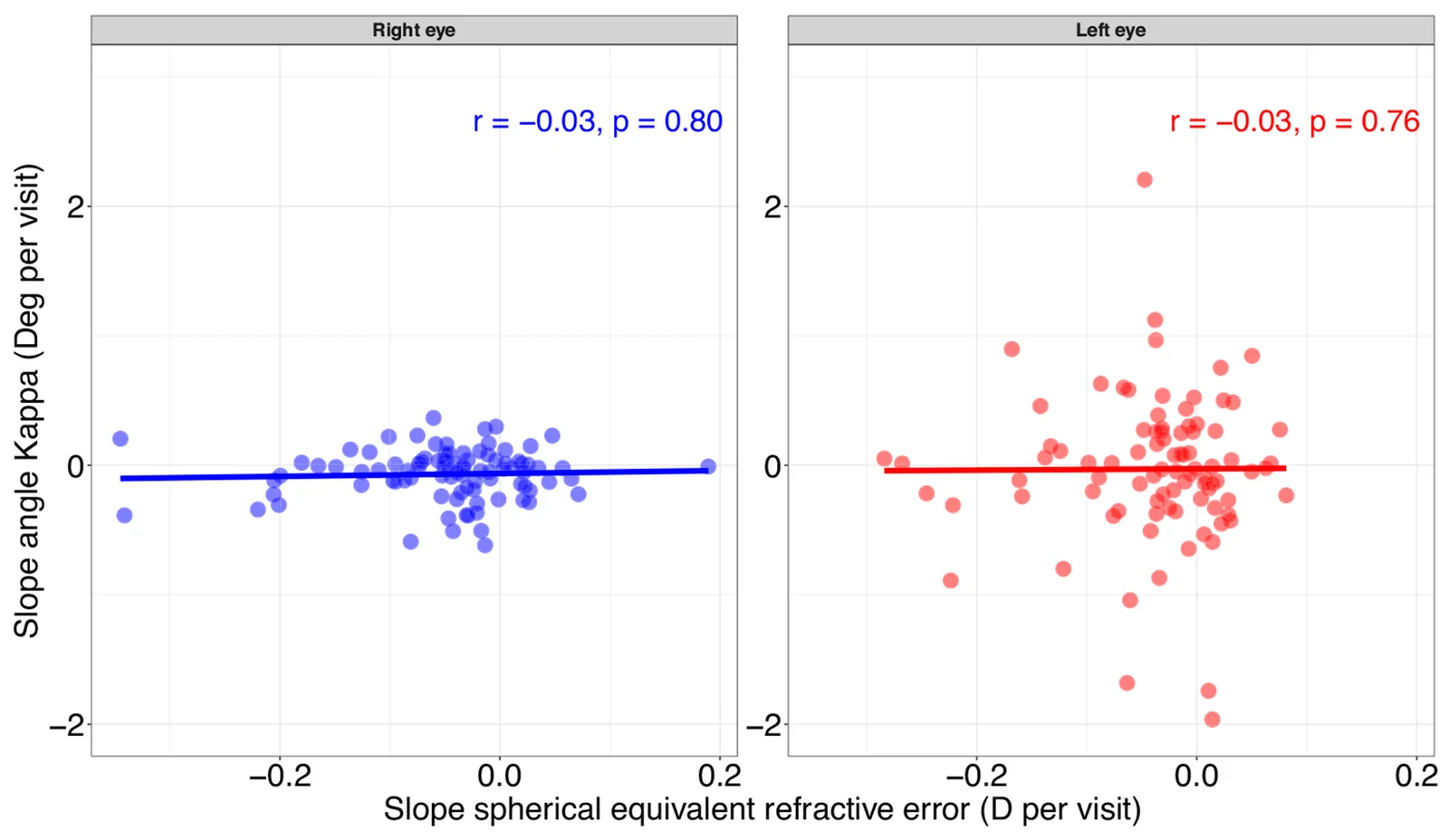
Scatterplot showing the slope of the magnitude of angle kappa (Deg, degrees per Visit), from Visit 1 through Visit 7, plotted across the slope of the spherical equivalent refractive error (M) for the right (blue dots) and left eyes (red dots). The slope of the angle kappa values did not show a significant correlation with the slope of spherical equivalent refractive error for either eye.

**TABLE 1:** Table summarising the biometric and angle kappa measurements.

	Baseline (Median ± IQR)		Slope (per visit)	
	Right eye	Left eye	Right eye	Left eye
M (D)	+1.00 ± 0.78	+0.92 ± 0.76	−0.04 ± 0.07	−0.02 ± 0.07

AXL (mm)	22.72 ± 0.71	22.71 ± 0.78	0.07 ± 0.04	0.07 ± 0.45

PCx (mm)	−0.28 ± 0.16	+0.29 ± 0.14	+7.82 × 10^−4^ ± 0.01	+14 × 10^−4^ ± 0.01

Angle kappa (degrees)	−4.97 ± 2.45	+5.32 ± 2.38	−0.03 ± 0.21	−0.03 ± 0.53

*Note*: Median and IQR are provided for PCx, AXL, M and angle kappa. Note the signs for PCx and angle kappa indicate the direction of the pupil barycenter in relation to the corneal apex (do not have numeric implication).

Abbreviations: AXL, axial length; M, cycloplegic spherical equivalent; PCx, pupil barycenter along the x-axis.

**FIGURE A1:**
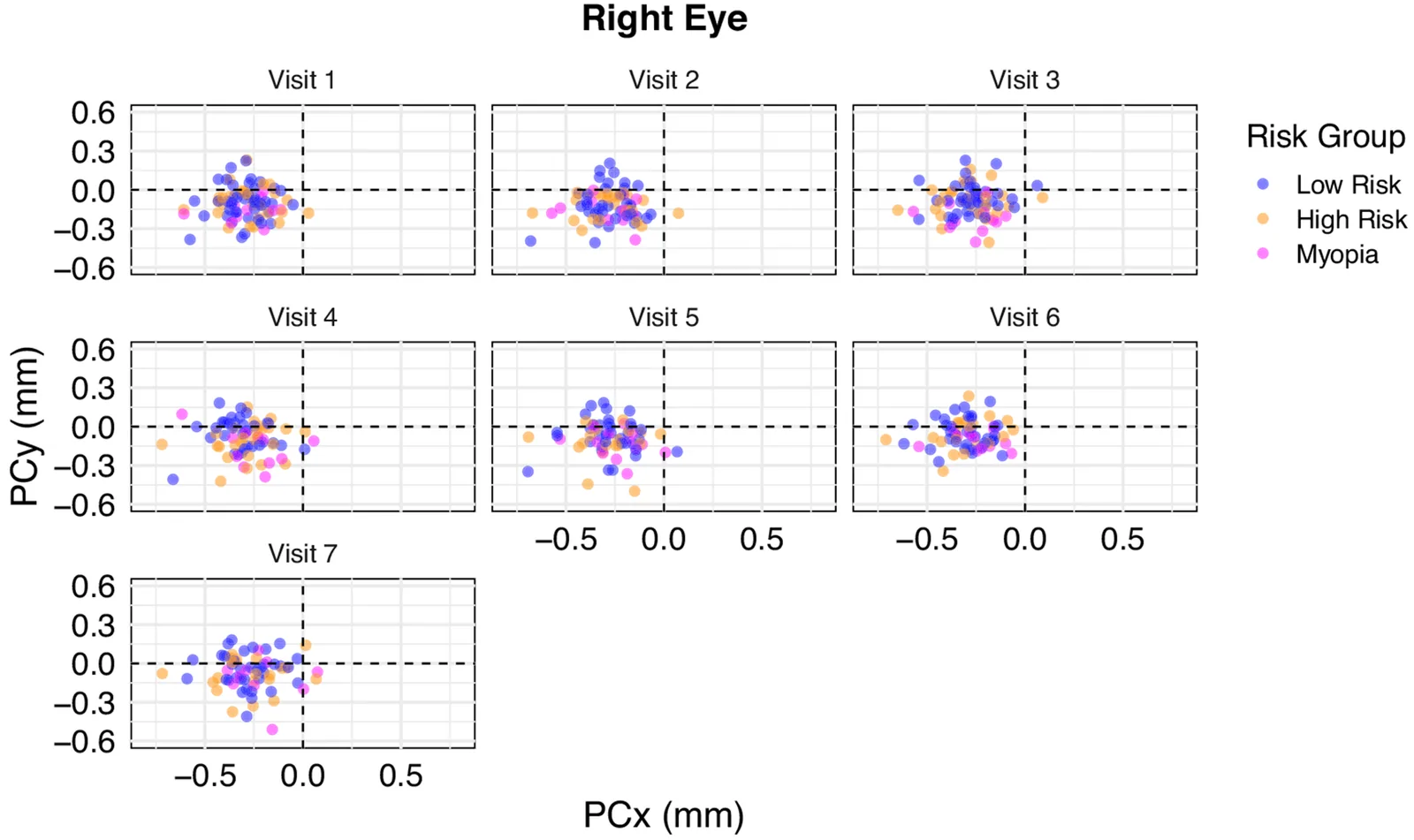
Pupil barycenter coordinate plot showing PCx across PCy coordinates relative to the corneal apex for the right eye for each visit. The blue dots show the low risk data, yellow dots show high risk data and the magenta dots show myopia data. There were no clear trends indicating differences in the groups at any visits. PCx and PCy are the horizontal and vertical distance from the centre of the entrance pupil to the corneal vertex, respectively.

**FIGURE A2:**
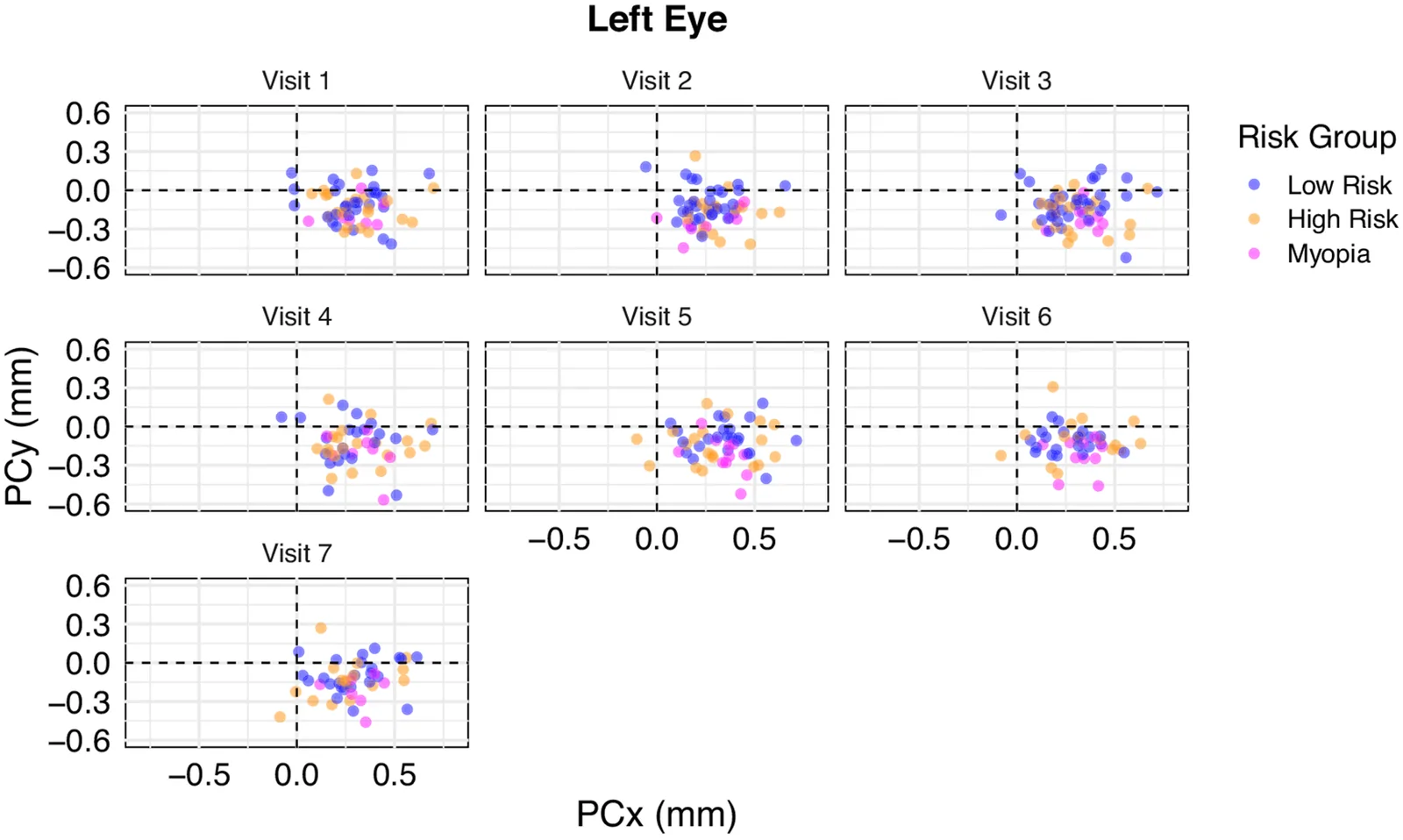
Pupil barycenter coordinate plot showing PCx across PCy coordinates relative to the corneal apex for the left eye for each visit. The blue dots show the low risk data, yellow dots show the high risk data and the magenta dots show myopia data. There were no clear trends indicating differences in the groups at any visits. PCx and PCy are the horizontal and vertical distance from the centre of the entrance pupil to the corneal vertex, respectively.

**FIGURE A3:**
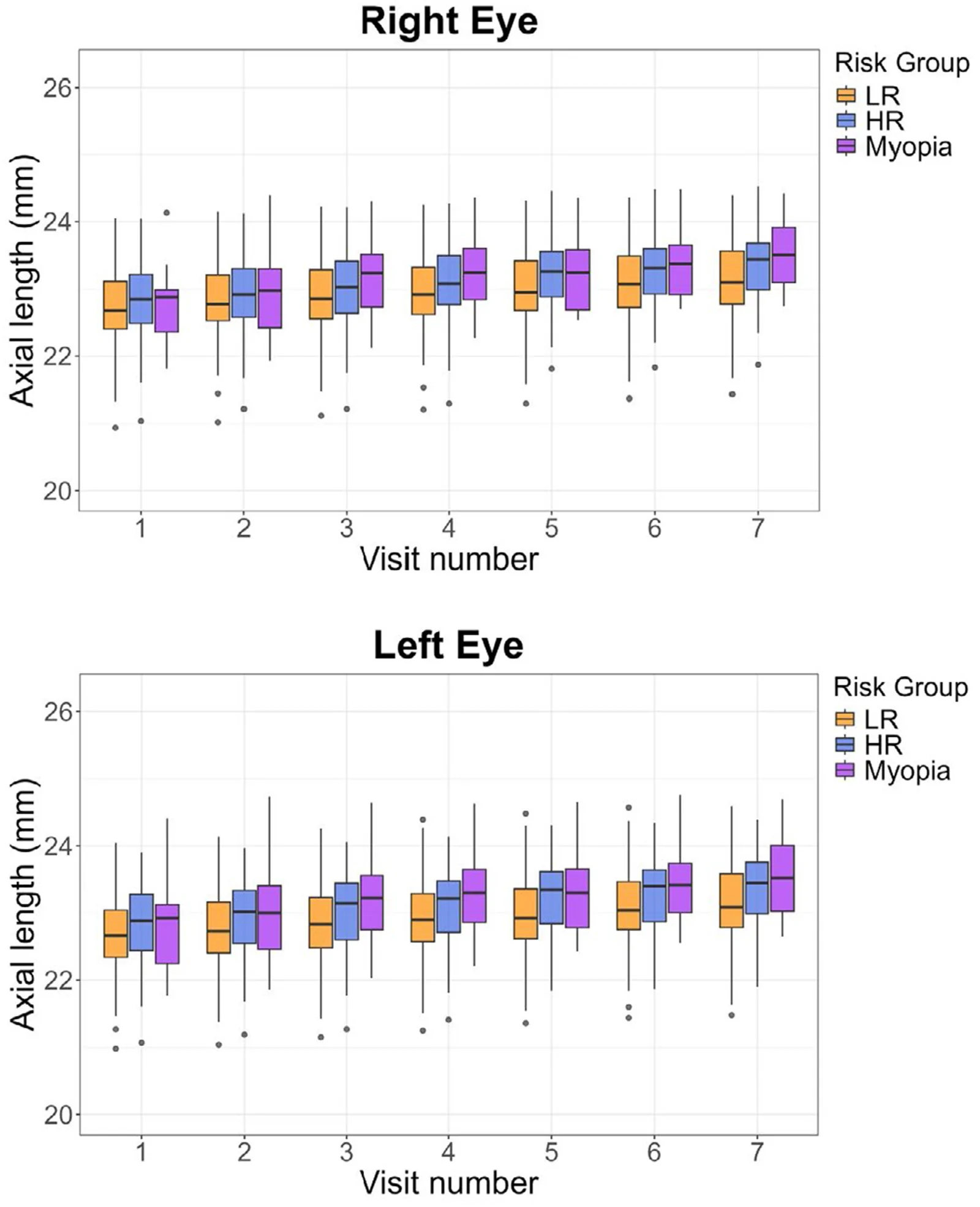
Boxplots showing the axial length results for each refractive group at each visit. Above: Right eye. Below: Left eye. HR, high risk; LR, low risk.

## Key points

- This is the first longitudinal study of the estimated angle kappa in young children (6 to ≤8 years at baseline).
- A commercially available optical biometer may be used to estimate angle kappa in young children.
- Angle kappa appears to be independent of axial length or refractive error in young children with functional emmetropia and a limited range of eye size, with a trend towards a smaller angle kappa in longer eyes.

## Apeendix A

**TABLE A1:** Specific inclusion criteria for the high and low risk groups used during recruitment. Spherical equivalent (M) values are presented for the right eye, as measured with cycloplegia.

Parental myopia	Age at baseline (years)	High risk	Low risk
One or two biological parents have myopia	6	+1.50 D ≥ M > −0.25 D	+2.50 D ≥ M > +1.50 D
	7	+1.00 D ≥ M > −0.25 D	+2.25 D ≥ M > +1.00 D
	8	+0.75 D ≥ M > −0.25 D	+2.00 D ≥ M > +0.75 D

None of the biological parents have myopia	6	+1.00 D ≥ M > −0.25 D	+2.50 D ≥ M > +1.00 D
	7	+0.75 D ≥ M > −0.25 D	+2.25 D ≥ M > +0.75 D
	8	+0.50 D ≥ M > −0.25 D	+2.25 D ≥ M > +0.50 D
